# Cancer Nano-Immunotherapy: The Novel and Promising Weapon to Fight Cancer

**DOI:** 10.3390/ijms25021195

**Published:** 2024-01-18

**Authors:** Daniel J. García-Domínguez, Soledad López-Enríquez, Gonzalo Alba, Carmen Garnacho, Carlos Jiménez-Cortegana, Rocío Flores-Campos, Luis de la Cruz-Merino, Nabil Hajji, Víctor Sánchez-Margalet, Lourdes Hontecillas-Prieto

**Affiliations:** 1 Department of Medical Biochemistry and Molecular Biology, and Immunology, School of Medicine, University of Seville, 41009 Seville, Spain; dgarcia-ibis@us.es (D.J.G.-D.); slopez9@us.es (S.L.-E.); galbaj@us.es (G.A.); cjcortegana@us.es (C.J.-C.); rflores-ibis@us.es (R.F.-C.); n.hajji@imperial.ac.uk (N.H.); 2 Institute of Biomedicine of Seville, IBiS, 41013 Seville, Spain; ldelacruzmerino@gmail.com; 3 Department of Normal and Pathological Cytology and Histology, School of Medicine, University of Seville, 41009 Seville, Spain; mgarnacho@us.es; 4 Oncology Service, Department of Medicines, School of Medicine, Virgen Macarena University Hospital, University of Seville, 41009 Seville, Spain; 5 Department of Medicine, University of Seville, 41009 Seville, Spain; 6 Cancer Division, Faculty of Medicine, Imperial College London, London SW7 2AZ, UK; 7 Clinical Biochemistry Service, Hospital Universitario Virgen Macarena, University of Seville, 41009 Seville, Spain

**Keywords:** cancer, immunotherapy, nanomedicine, clinical trials

## Abstract

Cancer is a complex disease that, despite advances in treatment and the greater understanding of the tumor biology until today, continues to be a prevalent and lethal disease. Chemotherapy, radiotherapy, and surgery are the conventional treatments, which have increased the survival for cancer patients. However, the complexity of this disease together with the persistent problems due to tumor progression and recurrence, drug resistance, or side effects of therapy make it necessary to explore new strategies that address the challenges to obtain a positive response. One important point is that tumor cells can interact with the microenvironment, promoting proliferation, dissemination, and immune evasion. Therefore, immunotherapy has emerged as a novel therapy based on the modulation of the immune system for combating cancer, as reflected in the promising results both in preclinical studies and clinical trials obtained. In order to enhance the immune response, the combination of immunotherapy with nanoparticles has been conducted, improving the access of immune cells to the tumor, antigen presentation, as well as the induction of persistent immune responses. Therefore, nanomedicine holds an enormous potential to enhance the efficacy of cancer immunotherapy. Here, we review the most recent advances in specific molecular and cellular immunotherapy and in nano-immunotherapy against cancer in the light of the latest published preclinical studies and clinical trials.

## 1. Introduction

Cancer is a multifaceted disease characterized by intricate cellular alterations and diverse molecular profiles, posing a significant challenge for effective treatment [1]. Carcinogenesis is a multistep process whereby the maintenance of cell proliferation, resistance of cell death, genomic instability, invasion, and angiogenesis are changes that occur in tumor cells triggered by genetic and epigenetic mechanisms [2,3]. Additionally, the interaction of tumor cells with the immune system and adjacent stroma allows the creation of a permissive environment supporting the tumor growth, invasion and metastasis, drug resistance, and immune evasion [1]. Therefore, providing an effective treatment is complicated given the complexity of this disease. Conventional treatments for cancer patients include chemotherapy (CT), surgery, and/or radiotherapy (RT) [4]. CT is being used as a first-line therapy for therapeutic purposes. Moreover, it is used to avoid tumor cells proliferation after surgery or radiotherapy or to reduce tumor size before surgery. The strategy and regimen for treatment depends on the type and the stage of cancer, significantly influencing patient survival outcomes. However, there are certain limitations such as the systemic toxicity, side effects, tumor recurrence, and resistance to treatment [5]. In order to address and overcome these challenges, new therapeutic treatments have emerged based on the better understanding of cancer pathogenesis.

Since the immunity is altered or blocked by tumor cells in most tumors, immunotherapy (IT) emerged as a promising alternative to cancer therapy, particularly regarding immune checkpoint inhibitors in treating solid tumors, which have improved survival and induced long-term survival and long-term durable remission [6]. Over the past years, the benefits of IT in cancer treatment have been demonstrated. However, although IT has advantages over conventional treatment [7,8], it still has some limitations. For example, efficacy, safety, or response rates are not satisfactory [9,10]; some IT agents have a very low solubility, poor stability, and short half-lives [11], and the delivery of immune agents through a tumor-permissive environment is not as effective as expected [12]. One strategy to overcome these problems is the use of nanoparticles (NPs). Thus, the combination of IT and nanomedicine, called nano-immunotherapy, can be employed as an efficient delivery system of immune agents while allowing the reduction in toxicity and the increase in solubility, stability, and half-lives of the agents. 

## 2. Immunotherapy: A Strategy to Enhance Anticancer Efficacy

**Figure 1:**
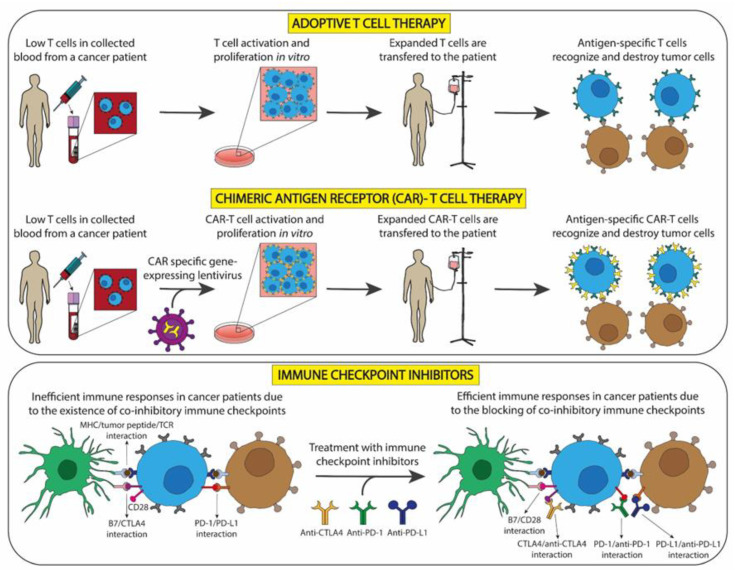
Therapeutic strategies involving T cells to treat cancer. **First box:** adoptive T cell and chimeric-antigen receptor T cell therapies, which do not need the use of drugs. **Second box:** immune checkpoint blockade, which requires the use of drugs.

The primary function of the immune system is to safeguard the organism against foreign and/or malignant own cells. In this sense, in 1950, Burnet M. and Thomas L. proposed “The Immune Surveillance Theory of Cancer”, which established that the immune system recognizes and destroys clones of transformed cells before they develop into tumors and kill tumors after they have been formed [13]. However, some transformed cells are able to evade immune defense mechanisms, allowing them to proliferate and form a tumor [14]. Although the immune responses are ineffective, these can be stimulated with treatments to destroy tumors, employing, for example, tumor antigens to induce a specific adaptive immune response. This immune reaction can prevent or limit the growth and spread of tumors, because tumors are known to trigger immune responses in their hosts. This innovative treatment modality is known as immunotherapy. Currently, immunotherapy is a growing field in which scientific evidence indicates that clinically important immune responses are related to T cells, which, after being activated by antigen presenting cells (APCs), such as dendritic cells (DCs), can specifically target the tumor microenvironment (TME). Among the advantages of IT over traditional antitumor therapy, there is the possibility of individualized treatment with minimal side effects and the prolongation of progression-free survival and overall survival [15,16]. Figure 1 illustrates various strategies employed in cancer treatment, including adoptive cell therapy, chimeric antigen receptor (CAR)-T cell therapy, and the inhibition of immune checkpoints.

### 2.1. Cellular Immunotherapy for Tumors

The identification of novel tumor antigens and methods of genetic modification of T lymphocytes to be selective against these antigens paved the way for a specific IT against tumors. This allows the differentiation between IT with non-genetically modified T cells or with genetically modified T cells [15].

#### 2.1.1. Immunotherapy with Non-Genetically Modified T Cells

The main method employed with tumor infiltrating lymphocytes (TILs) is an adoptive cell therapy, which consists of extracting lymphocytes from the tumor place and culture them in vitro while adding different stimuli, such as IL-2, and finally infusing them in the same patient (autologous treatment) [17]. This easy method is an effective therapy option, because these specific cells are able to recognize various tumor antigens rather than a single antigen [18] with a 40–50% response in patients with melanoma [19,20]. This method could be relevant in tumors where the presence of TILs has been established as a strong predictor of a favorable prognosis [21]. Currently, the practical perspective of TILs in different cancer types is promising for the use of monoclonal antibodies, such as antiprogrammed cell death (PD)-1 and anti-CD137 in the selection of T cells in TIL cultures with CD3+, helper, and cytotoxic T cell (CD4+ and CD8+ cells, respectively) as different fractions of TILs represented in the TME [22]. Moreover, this method can be improved with a rapid standardized expansion protocol [23] and the use of different co-stimulatory signaling pathways (e.g., TNF-R, 4–1BB, and CD-137) to improve CD8+ effector-memory T cells expansion [24,25]. 

The treatment of TILs can be enhanced with other co-stimulatory molecules, such as IL-2 (previously mentioned), which has demonstrated superior relapse-free survival time [22,26,27,28]. Deninger et al., in a pilot study, showed interesting results with the *v-raf* murine sarcoma viral oncogene homolog B1 (best known as *BRAF*) inhibitor combination, where they observed that seven out of thirty-five patients had an objective therapeutic response, and two of them had a sustaining response that lasted up to three years in metastatic melanoma [29] or with anticytotoxic T lymphocyte-associated molecule-4 (CTLA-4) drug ipilimumab in high-risk, locally/regionally advanced melanoma [30]. However, the inhibition of the downstream mitogen-activated extracellular signal-regulated kinase (MEK) blocked T cells, reducing the expectations [31,32]. Currently, researchers are exploring the co-stimulation of TILs with anti-PD-1 to prevent T cell exhaustion [33], but clinical trials were based only on the use of anti-PD-1, pembrolizumab, which ended up causing intense infiltration of TILs naturally [34]. In this line, recent clinical trials with TILs are currently being carried out with promising results in breast cancer (BC) [35], lung cancer [36], and ovarian cancer [21].

Another method is the use of endogenous T cells, which consists of isolating antigen-specific T cells from peripheral blood [37]. This approach boasts several advantages over the aforementioned method. Endogenous T cells are more readily accessible and require lower doses of interleukin (IL)-2, are not limited to the tumor site, and present less exhaustion and less cytotoxicity [38,39,40,41,42]. This method presents the complexity of isolating antigen-specific cells because very few cells are obtained, in addition to the need for producing more immunogenic targets. For this reason, APCs are necessary, such as DCs. DCs may be modified to express the desired antigens [43,44,45]. Heterologous DCs to the patient can be used, such as the K562 cell line [46], but the most used are autologous DCs [40,47]. Using peptide-major histocompatibility complex (MHC) tetramers or quantitative assessments of a specific region of the T cell receptor (TCR, also known as CDR3) that recognizes the MHC/epitope of interest can increase the frequency of tumor-reactive cells [48]. Co-stimulation of T cells with IL-21 resulted in a ten-fold increase in the frequency of tumor-associated antigen (TAA)-specific CD8 T cells [49]. Moreover, the depletion of CD25 promoted a remarkable 100- to 300-fold increase in the number of TAA-specific T cells [50]. Although previously it was virtually impossible to isolate New York esophageal squamous cell carcinoma 1 (NY-ESO-1)-specific T cells from seronegative donors, it is now feasible to isolate such cells from both cancer patients and healthy donors [51]. The effects of NY-ESO-1-specific redirected T cells are currently been studied in clinical trials, but in this case, they are genetically modified to target these intracellular antigens [52].

#### 2.1.2. Immunotherapy with Genetically Modified T Cells

The objective of this method is to redirect the antigen specificity of T cells by stable genome integration or transient RNA electroporation using viral (retroviral or lentiviral transduction) or non-viral (transposon/transposase system) approaches [53,54,55]. Currently, there are two different systems:

(a) T cell receptor (TCR): Through genetic engineering, a population of T cells with high expression of TCR specific for TAA has been created. These T cells promote the antitumor response and allow the expression of TCR variants with higher affinity for tumor recognition [56,57]. The first trials were performed by TCRs modified with melanoma-associated antigen recognized by T cells (MART-1) from TILs of patients with malignant melanoma. In these trials, a lower response (~13%) has been verified compared with therapies with TILs, but it could be used in patients with poor responses to TILs treatments as previously suggested [58]. Nevertheless, the most appropriate antigen seems to be NY-ESO-1 according to the accumulation of more than 36 different clinical therapies with response data of 61% to 50% in synovial sarcoma and melanoma patients [59,60]. Phase I/II trials investigating NY-ESO-1/LAGE-1 TCR-engineered T cells in advanced multiple myeloma following autologous stem cell transplantation resulted in an 80% clinical response rate. Thus, T cells were safe, trafficked to the marrow, and demonstrated prolonged persistence associated with clinical activity [61]. More recently, there have been other clinical trials with this type of genetically modified cells [52].

However, it has been described that these antitumor responses induced by other TCR variants showed toxicity in patients. These effects were observed when directing TCRs towards testicular cancer antigens, such as the melanoma antigen gene (MAGE) family, which presented serious neurotoxicity problems in three of eight patients treated with TCR MAGE-A3/A9 [62] and cardiac toxicity due to treatment with TCR MAGE-A1 [63,64]. These effects were caused by cross-reactivity that could be prevented with peptide homology prediction models. 

(b) Chimeric antigen receptors (CAR-T): CAR-T cells are T cells modified ex vivo to express a chimeric receptor with an antigen receptor containing a single-chain variable fragment (scFv) and an intracellular TCR signaling domain. The scFv is the target tumor cell-directed recognition domain. The intracellular domain of CAR contains several components with CD3-ζ (first generation) [65] in addition to a co-stimulatory domain such as CD28 or 41bb (second generation) or both (third generation) [66]. Fourth-generation CAR-T cells are known as armored CAR-T cells that co-express key cytokines to improve the efficacy and suicide genes for the safety of CAR-T therapy [67,68,69]. Particularly, recognition of CAR-T target cells does not require human leukocyte antigen presentation. For this reason, autologous T cells from cancer patients are isolated and genetically modified to express CAR, redirecting the T cells specificity to TAA. Thus, adoptively transferred CAR-T cells are equipped to induce and maintain remissions through a synergy of antibody-based target cell recognition and effector and memory function of T cells. Thus, there are receptor synthetics that allow T cells to identify TAA independently of MHCs and to identify non-peptide antigens, thus preventing tumor escapes through low MHC expression [70]. These results represent a substantial improvement compared to conventional therapies that produce a complete and durable response rate.

The most advanced CAR therapies are those directed at B cell malignancies. These treatments are tisagenlecleucel and axicabtagene ciloleucel. Tisagenlecleucel is a CAR made up of a murine anti-CD19 single-chain antibody fragment fused with intracellular signaling domains of 4-1BB (CD137) and CD3ζ. The CD3ζ component is critical for initiating T cell activation and antitumor activity, while 4-1BB enhances the expansion and persistence of tisagenlecleucel. Axicabtagene ciloleucel is also made up of an anti-CD19 and CD3ζ but with a co-stimulatory CD28 domain. Both treatments produced a reprogramming of the T cells of patients with a transgene that encodes a CAR to identify and eliminate cells that express CD19 [71,72,73].

There are other CAR treatments for other types of liquid tumors, such as natural killer group 2D (NKG2D) ligand-targeting therapies for acute myeloid leukemia, since NKG2D ligands are widely expressed in many malignancies and preclinical experiments with the respective CAR constructs showed promising results [74,75]. Additionally, treatments against different ligands are also showing some promising results, such as C-type lectin-like molecule 1 (CLL-1) [76], FMS-like tyrosine kinase 3 (FLT3) [77,78,79], CD33 [80,81] or CD123 [82,83]. However, these treatments may promote a variety of complications, such as the cytokine release syndrome, the most common adverse effects of CAR T cell therapy are high fever, low blood pressure, and hypoxia, with or without toxicity in multiple organs, including cardiovascular, gastrointestinal, respiratory, renal, hematological, and nervous systems. The trigger for this condition is the activation of T cells in the interaction of their TCRs or CARs with cognate antigens expressed by the tumor cell [84]. Other complications detected are neurological events or myelosuppression [85].

In general, the main positive results of these therapies have been found in liquid tumors, and the great challenge of these treatments is against solid tumors. There are many reasons why CARs do not function properly in solid tumors, such as defective transfer of these cells to the tumor site, poor grafting, and expansion of CAR cells [86]. This may be improved with co-expression of the C-C motif chemokine receptor (CCR)2b [87] and the hostile microenvironment of the tumor [88]. The latter seems to be the most decisive for solid tumors due to its ability to generate immunosuppression driven by necrosis, lack of nutrients, hypoxia [89], depletion of T cells through PD-1 [90], and other immunosuppressants agents, such as transforming growth factor (TGF)-β and IL-10 [89]. Some research groups have shown clear improvements in the efficiency of these treatments with CAR cells co-stimulated with anti-PD-1, as shown in glioblastoma with a CAR-T anti-EGFRvIII therapy [89].

Currently, treatments with CAR-T cells are in the experimental phase with different approaches. In this line, image-guided insertion of these T cells is being tested in patients with pleural malignancies [91] or in combination with the anti-PD-1 agent pembrolizumab [92]. Even though, in this type of tumor, the search of an adequate, specific TAA that directs the immune reaction towards the tumor cells and causes minimal damage to healthy cells is very complex, unlike what occurs in hematological neoplasms with CD19, and the solution of this problem will be in the numerous clinical trials with such different approaches [93].

## 3. Nanomedicine in Cancer

Nanomedicine, defined as the use of nanotechnology for diagnosis, monitoring, control, prevention, and treatment of diseases [94], has been exploited in the last decades to overcome limitations of traditional chemotherapeutic agents. Among the major drawbacks of CT that limit its clinical significance in cancer treatment is the lack of drug-specific affinity for cancer cells. As a result, it is necessary to administer the chemotherapeutic agent in large quantities to achieve a therapeutic concentration in the tumor, leading to non-specific toxicities and adverse side effects. Additional critical issues include low bioavailability, poor water solubility, uncontrolled release, and difficulty of drugs to cross biological barriers [95,96]. To overcome these challenges, nanomedicine strives to minimize anticancer drug degradation and inactivation upon administration, prevent undesirable side effects due to high doses, and increase delivery of the active drug into cancer cells.

The design of drug delivery systems for cancer treatment must consider a number of challenges that these nanomedicines must overcome in order for the treatment to be effective. Once in circulation, nanomedicines can be recognized by the cells of the mononuclear phagocyte system and cleared from circulation. At the next level, they must successfully extravasate from the circulation to reach the tumor, navigating the tumor microenvironment (TME) characterized by a dense interstitial matrix, low pH, low oxygenation, and high interstitial fluid pressure. Lastly, since most anticancer drugs target the cytoplasm or specific cell organelles, it is essential for these nanomedicines to efficiently cross the plasma membrane through various mechanisms [97]. Advances in nanotechnology enable the design of multifunctional nanomedicines with the necessary attributes to overcome both biological and pathological barriers.

### 3.1. Nanocarriers for Cancer Treatment

Nanotechnology-based delivery systems utilize nanocarriers for drug transportation, which are macromolecular assemblies with a submicron particle size, typically less than 500 nm, capable of incorporating the therapeutic agent through encapsulation or covalent linkage to the carrier surface [96,98]. Due to their small size, nanocarriers possess a high surface-to-volume ratio that makes them have a high drug loading capacity [95]. Over the last years, the nanocarriers that have been more extensively used range from 10 to 100 nm diameter, since that size range reduces renal clearance. Nanocarriers larger than 100 nm in size greatly change their biodistribution and pharmacokinetic properties, and they are cleared by the reticuloendothelial system.

Various types of nanocarriers are under investigation for cancer treatment, acknowledging that their composition, size, shape, and surface properties can modify the physiochemical properties of the nanocarrier [96,98]. They can be synthesized from various organic, inorganic, or biological material and can be classified into several groups based on the components used for synthesis or the structural aspects of the nanocarrier. Each of these groups have numerous advantages and disadvantages regarding drug loading capacity, stability, and delivery [99]. 

#### 3.1.1. Inorganic Materials

Inorganic materials have been used to synthesize nanocarriers such as gold NPs and nanorods, silver nanostructures, iron oxide NPs, mesoporous silica-based NPs, carbon-based nanomaterials, and quantum dots. The unique physical, magnetic, electrical, and optical properties of inorganic nanocarriers make them highly valuable for diagnostics and imaging applications. For example, gold (AU) NPs have been used to label primary human T cells in order to track them and study the fate of immune cells in cancer immunotherapy, specifically in melanoma [100]. However, the low solubility and potential toxicity of inorganic materials limit their use in clinical practice. Another challenge that limits the use of inorganic nanocarriers in cancer drug delivery systems is that, if they are not modified, the drug must be bound onto the surface of the nanocarrier. Thus, the therapeutic agent is not protected from degradation during transport, and their impact on untargeted cells cannot be avoided [96].

#### 3.1.2. Organic Materials

Organic materials used to design nanocarriers include natural and synthetic polymers, most of them biodegradable and biocompatible. Commonly employed natural polymers include proteins such as human serum albumin, lipids, and polysaccharides like chitosan, that are being used to form NPs and liposomes. Undoubtedly, lipid-based nanocarriers, particularly liposomes, are the most widely used cancer drug delivery systems, due to their simple formulation and properties such as biocompatibility, bioavailability, self-assembly, and high drug loading capacity. Additionally, liposomes have the capacity to accommodate hydrophilic agents in their aqueous core and hydrophobic drugs in the lipid bilayer. For these reasons, a majority of Food and Drug Administration (FDA)-approved nanomedicines are lipid-based nanocarriers [99,101]. For instance, Doxil (a pegylated liposomal NP of doxorubicin) was the first NP approved by the FDA for ovarian cancer, acquired immune deficiency syndrome-related Kaposi’s sarcoma, and multiple myeloma [102]. This nano-formulation acts against tumor cells and induces an antitumor immune response by eliminating myeloid-derived suppressor cells (MDSCs) and enhancing the efficacy of adoptive T cell transfer in BC [103].

In recent decades, polymeric nanoparticles have gained great attention due to several promising features they show as cancer drug delivery systems. They can be synthesized from natural or synthetic material, and based on their composition and structural organization, they form a wide variety of nanostructures, such as polymersomes, micelles, or dendrimers [99,104]. Polymeric NPs present high loading capacity, stability, and controlled release profile. In addition, their surface can be easily modified for additional targeting. Although showing promising characteristics for cancer drug delivery, potential toxicity of polymer NPs and particle aggregation also raises serious safety concerns [101,104]. 

#### 3.1.3. Biological Materials

In recent years, interest has increased in nanocarriers made of biological materials, obtained directly from a biological source, or synthesized to mimic nanostructures of biological origin. Extracellular vesicles, including microvesicles and exosomes; virosomes, liposome-like structures with special glycoproteins that allow improved targeting; bacterial minicells and bacterial vesicles; and virus-like particles, are being tested for the delivery of anticancer drugs [96,105,106]. An exosomal doxorubicin (Exo-Dox) has been used against cancer cells. Exo-Dox inhibited the growth of the Michigan Cancer Foundation (commonly known as MCF)-7 BC cells in vitro and in vivo [107]; and Exo-Dox also increased the efficacy of doxorubicin alone in breast and ovarian cancer mouse tumors [108]. Furthermore, exosomes loaded with α-galactosylceramide and ovalbumin activated the adaptive immunity (NK, CD8+ T cells or gamma-delta T cells) in order to inhibit tumor growth [109].

### 3.2. Targeting Strategies

**Figure 2:**
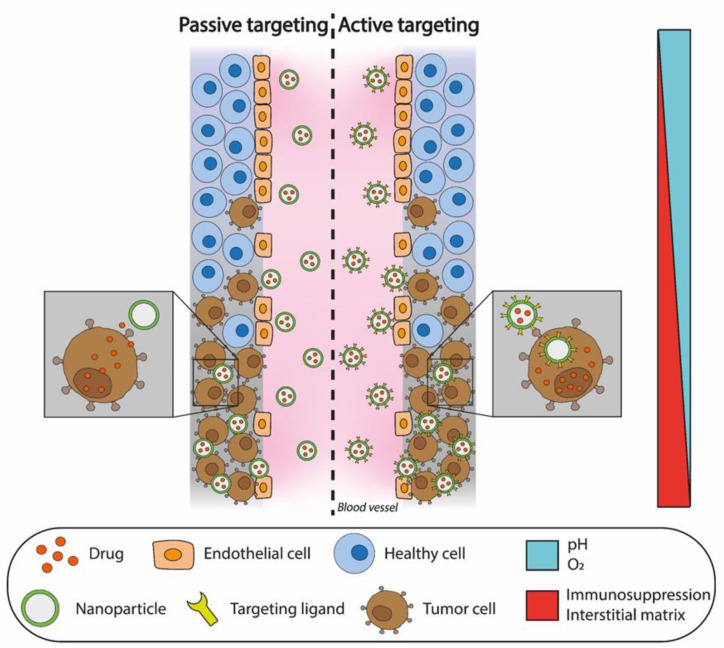
Targeting strategies to treat cancer in nanomedicine. Once in circulation, drug-containing nanoparticles go through the blood vessels to target tumor cells in a passive manner (**left**), following the enhanced permeability and retention effect, or in an active manner (**right**), in which nanoparticles have previously acquired specific targeting ligands to interact with tumor cells.

Anticancer drug-loaded nanocarriers must overcome several barriers before reaching tumor cells. In addition to that, the accumulation of anticancer drug-loaded nanocarriers in the tumor is necessary to obtain the desired therapeutic effect. Several strategies have been developed to overcome both physiological and pathological barriers, including passive and active targeting [110,111], as shown in Figure 2. 

#### 3.2.1. Passive Targeting for Cancer Treatment

Drug targeting strives for selective and effective localization of the active drug at the specific target organ, tissue, or cell where a specific pharmacological impact is required while restricting its access to untargeted normal cells, thus minimizing toxic effects. The main mechanism of tumor targeting of anticancer nanomedicines is based on the Enhanced Permeability and Retention (EPR) effect, first described by Matsumura and Maeda in 1986 [112] as a passive yet powerful targeting strategy. They showed that tumor tissues had defective blood vessels with aberrant branching and large openings between endothelial cells of around 400 nm due to their rapid proliferation and decreased number of pericytes. This unique characteristic helps macromolecules, such as nanocarriers, easily move into tumor tissues after crossing the endothelium barrier. Furthermore, solid tumors have a poor lymphatic drainage system, leading to extended retention times of nanocarriers in the tumor extracellular matrix. 

Although the EPR effect has allowed the successful development of some anticancer nanomedicines with a passive tumor-targeting mechanism [99], many well-designed drug delivery systems fail when tested in patients. Over the last years, the role of the EPR effect has been investigated, focusing on the influence of the TME, heterogeneity of human tumors, and differences between animal models and actual patients [104,113,114]. These factors may contribute to the observed limited penetration of nanomedicines in human tumors, highlighting the need for further refinement of passive targeting strategies.

#### 3.2.2. Active Targeting for Cancer Treatment

To improve the efficacy of tumor targeting, the most extensive approach has been to functionalize the nanocarrier’s surface with a targeting ligand, a molecule with a great affinity to highly expressed receptors on cancer cells, providing a more significant accumulation of nanocarriers in the tumor [96,98,104]. Many active targeting ligands are being tested for cancer therapy, including low molecular-weight ligands such as glucose, folate, or transferrin, antibodies and their fragments, aptamers, peptides, polysaccharides, or nucleic acids [95,101]. It is well known that many epithelial cancers overexpress folate receptors, hyaluronic acid receptors, or transferrin receptors, therefore they are the target of effective CT. Unfortunately, some of these receptors are also expressed in healthy tissues, limiting the therapeutic efficacy [95]. 

As mentioned earlier, most anticancer drugs exert their effect inside the cell, thus it is essential to design nanocarriers capable of crossing the plasma membrane. Active targeting of nanocarriers is a promising approach since binding of the targeting ligand to its cell membrane receptor may induce endocytosis [98]. 

A different approach to active targeting is to use nanocarriers targeted to the vasculature or microenvironment instead of tumor cells. For instance, targeting moieties on the vascular wall, including vascular endothelial growth factors (VEGFs), vascular cell adhesion molecules (VCAMs), matrix metalloproteases (MMPs), and αvβ3 integrins, leads to destruction of endothelial cells of tumor vessels. Consequently, blockage of oxygen and nutrients supply leads to the death of tumor cells [115]. Active targeting is still evolving, but its potential to revolutionize cancer treatment is undeniable. By precisely delivering drugs to the right place at the right time, we can maximize therapeutic impact while minimizing collateral damage. The future of cancer therapy is looking brighter, one targeted ligand at a time.

#### 3.2.3. Smart Nanomedicines

An ideal cancer drug delivery system should have the ability to not only target cancer cells specifically but also control drug release so that it only occurs once it reaches the tumor. Controlled drug release leads to a higher therapeutic index and minimizes undesired side effects [97]. In that regard, researchers have developed in recent years stimuli-responsive nanomedicines, capable of site-specific release of the anticancer drug into the tumor.

The TME has a lower pH than normal tissue, due to the high rate of glycolysis in tumor cells. Some nanomaterials behave differently at different pH, a beneficial feature that can be used to develop pH-sensitive nanomedicines. For instance, Liao et al. developed a doxorubicin-loaded carrier of hyaluronic acid using hydrazone linkages that can be cleaved in an acidic environment, showing enhanced release of doxorubicin into the tumor [116,117]. The reducing environment of cancer cells can also be used for the development of redox-responsive nanomedicines. For example, Bansal and co-workers conjugated doxorubicin on hydroxyl-terminated polymer (mPEG-b-PJL-OH), which exhibited high efficacy and low cytotoxicity owing to its redox-responsive disulfide bond [118].

Additional common stimuli responses such as photo, heat, magnetic field, or endogenous stimuli such as some enzymes in cancer cells can be used to design smart nanomedicines capable of triggering drug release as required [96,119]. 

Despite well-designed cancer nanomedicines, several studies have shown that the amount of NPs capable of penetrating a solid tumor is not sufficient for the treatment to be effective [120,121]. To deal with this crucial issue, several researchers have developed strategies to degrade collagen using different agents [122,123,124,125].

It is worth noting that significant progress has been made in recent years in the development of anticancer nanomedicines, as evidenced by the large number of them that are in different phases of clinical trials [126]. However, it is necessary to continue working to overcome the challenges posed by the biological and pathological barriers of the tumor, as well as the defense mechanisms of the immune system.

## 4. Nano-Immunotherapy: A Reality in Cancer Treatment

Nano-immunotherapy is the combination of nanomedicine and IT. In recent years, ever more nano-formulations have been used to improve the efficacy and minimize side effects [127,128] of cancer IT. Therefore, the integration of both has become a topic of widespread interest in the field of cancer. 

The application of NPs in cancer IT can be simplified in two different targeting strategies:

(a). Nano-immunotherapy against tumor cells: CT drugs (e.g., doxorubicin, oxaliplatin, and paclitaxel), RT, photodynamic and photothermal therapies, as well as other physical stimuli are able to induce immunogenic cell death [127,128,129,130]. Examples of this kind of treatment are:
  - Oxaliplatin: This CT drug encapsulated in monomethoxy-poly(ethylene glycol)-poly (d,l-lactide-*co*-glycolide (PLGA-mPEG) NPs (OXA-NPs) triggered more damage-associated molecular pattern (DAMP) release and induced a stronger DC and T lymphocyte infiltration and activation in pancreatic tumor cells in vitro than oxaliplatin. Moreover, OXA-NPs inhibited tumor growth in immunocompetent mice, exhibiting stronger therapeutic effects than the OXA group [131]. Similarly, magnetic NPs, as a delivery system of oxaliplatin, reinforced immunogenic cell death induction of that CT drug [132]. Additionally, the combination of oxaliplatin and PD-L1 trap fusion protein using liposomal NPs inhibited tumor growth in an orthotopic colorectal mice model and showed T cell activation [133].
  - Paclitaxel: 1-NP, a paclitaxel NP, exhibited much lower cytotoxicity to macrophages than did PTX at the same high PTX concentration and maintained the capacity to stimulate macrophages to polarize into M1 and inhibit their M2 differentiation both on phenotypical and functional levels in a dose-dependent in vitro and in melanoma tumor-bearing mouse model [134]. Moreover, paclitaxel and SP-LPS, a Toll-like receptor (TLR) 4 agonist, were encapsulated into a bio-polymer, and their combination increased chemotherapeutic and immunotherapeutic activity both in vitro and in vivo as compared to the paclitaxel-treated group in melanoma [135].
  - Doxorubicin: Doxorubicin was integrated into mesoporous silica NPs (DOX@HIMSNs) for a systemic treatment of triple negative BC (TNBC). DOX@HIMSNs enhanced antitumor efficacy and induced DC maturation and antitumor cytokine release as compared with doxorubicin [136]. Gao et al. developed an NP in which doxorubicin was conjugated with anionic polymer hyaluronic acid via a tumor overexpressed matrix metalloproteinase sensitive peptide [137]. This NP combined with anti-PD-1 led to better results in vivo, improving the antitumor efficiency [137]. Moreover, doxorubicin and recombinant human IL-2 were co-delivered in hydrophilic cationic polymer NPs [138]. This combination delayed tumor growth and increased tumor-infiltrated cytotoxic T lymphocytes in a hepatocellular carcinoma model [138].
  - Radiotherapy: The combination of localized radiation with NBTXR3 (a radio-enhancing NP) and systemic anti-PD1 treatment in a lung cancer mouse model was explored. This combination significantly delayed tumor growth in anti-PD1-resistant and -sensitive metastatic lung cancer cells, which could open the possibility of its use to treat patients with metastatic lung cancer regardless of their sensitivity (or resistance) to immunotherapies [139]. Additionally, PLGA-R837@Cat NP is an NP based on poly (lactic-co-glycolic) acid (PLGA) loading with hydrophobic imiquimod (R837), a TLR-7 agonist, and water-soluble catalase (Cat) [140]. These NPs have enhanced RT efficacy, inhibited tumor metastases, and induced antitumor immune responses [140].
  - Photothermal therapy (PTT): In neuroblastoma, Cano-Mejia et al. developed a combination of nano-immunotherapy and PTT, which resulted in CpG oligodeoxynucleotide-coated Prussian blue (CpG-PB) NPs. PPT triggered tumor cell death and released TAA. Immunogenicity increased in vitro, and a high survival and a complete tumor regression of 70% at 60 days in treated mice was observed with this combination in a syngeneic neuroblastoma mouse model [141]. Subsequently, the same authors used this combination with anti-CTLA-4 in neuroblastoma, obtaining similar results. Additionally, an alteration of the surface levels of co-stimulatory, antigen-presenting, and co-inhibitory molecules on neuroblastoma cell lines resulted with the use of an anti-CTLA-4 therapy [141]. In BC, ovalbumin-coated PEGylated MnFe2O4 NPs loaded with R837 immunoadjuvant (R837-OVA-PEG-MnFe2O4) NPs were used together with PTT. These NPs downregulated M2-associated cytokines, tumor growth was inhibited, and lung metastasis was prevented [142].
(b). Nano-immunotherapy against the TME: NPs can be used to modulate TME by inhibiting immunosuppression or by increasing the immune system activation.
  - Inhibition of immunosuppression: The inhibition of immunosuppression in cancer has been evaluated with different nano-formulations, particularly affecting immune cells. For example:
    - Regulatory T cells (Treg): PEG-modified single-walled carbon nanotubes (PEG-SWCNTs) against specific receptors of Tregs, which are highly found within the TME, were designed by Sacchetti et al. [143]. In vivo assays showed that Tregs, residing in the melanoma TME, uptake PEG-SWCNTs more efficiently than intratumor non-Tregs or splenic Tregs [143].
    - Macrophages: In vitro co-cultures with adenocarcinoma cell lines, macrophages, and ferumoxytol (an iron oxide NP) increased pro-inflammatory Th1-type responses. In vivo, ferumoxytol reduced tumor growth of subcutaneous adenocarcinomas in mice and increased pro-inflammatory M1 macrophages in tumor tissues [144]. Lastly, Glycocalyx-mimicking NPs (GNPs) self-assembled into amphiphilic copolymers to target tumor-associated macrophages (TAMs) in Lewis lung cancer were evaluated [145]. GNPs were internalized by TAMs via lectin receptors, which resulted in a phenotypic change towards M1 macrophages. Additionally, GNPs reduced tumor growth by depleting Tregs, and its combination with anti-PD-L1 increased IL-12 levels, decreased IL-10, arginase, and C-C motif chemokine ligand (CCL) 22 in tumor-induced mice [145].
    - MDSCs: MDSCs are predominantly polarized into M2 in BC. However, two cationic polymers (cationic dextran and polyethyleneimine) were able to repolarize M2-MDSCs into the M1-type. In a BC mouse model, intratumoral administration of these NPs reduced both tumor growth and the percentage of tumor-induced MDSCs in blood, spleen, tumors, and bone marrow [146]. Moreover, both cationic polymers promoted the proliferation and activity of CD4+ and CD8+ T cells in vivo, which showed that restoring T lymphocyte function in the tumor environment is critical because it effectively induces massive necrosis of tumor cells [146]. The reduction of the MDSC activity using synthetic high-density lipoprotein-like NPs (HDL NP) specifically binding the scavenger receptor type B-1 (SCARB1) has also been studied. These NPs reduced tumor growth and metastatic tumor burden, as well as increased CD8+ T cells and reduced Tregs in the metastatic TME in a melanoma mouse model [147].
  - Activation of immune responses: Anti-immunosuppressive factors can be delivered by NPs to the TME in order to enhance the immune system activation. Thus, TGF-β inhibitor and IL-2 were co-delivered to the TME by nanoscale liposomal polymeric gels (nLGs) [148]. An increase in the number and activity of NK cells and CD8+ T cells was observed together with a reduction in tumor growth in a melanoma mouse model [148]. Similarly, Xu et al. used liposome-protamine-hyaluronic acid (LPH) NPs loaded with TGF-β small interfering (si)RNA promoting TGF-β downregulation in melanoma. This inhibition increased tumor-infiltrating CD8+ T cells, decreased Tregs, and inhibited tumor growth [149]. Inhibitors of metabolic enzymes have also been evaluated due to their participation in the immune modulation. NPs loaded with the Indoleamine 2,3-dioxygenase 1 (IDO) inhibitor 1-MT modified with hyaluronic acid and with an anti-PD-1 antibody showed an increment of T cell immunity and an immunosuppression reduction, which resulted in a beneficial antitumor effect in melanoma-bearing mice [150]. Furthermore, IDO inhibitor and oxaliplatin were conjugated in mesoporous silica NPs. This combination caused tumor reduction or eradication, as well as an increase in the recruitment of CD8+ T cells along with a reduction in Foxp3+ T cells in a pancreatic ductal adenocarcinoma mouse model [151].

Another described strategy was the activation of T cells. To this end, phytohemaglutinin was encapsulated in a liposome-improved T cell activation in a variety of tumors, including (but not limited to) multiple myeloma, leukemia, glioma, breast, and lung cancers [152]. This NP activated T cells in vitro following 24 h of treatment, and these T cells killed all tumor cells regardless of the tumor type [152]. Other NPs to deliver CTLA-4-siRNA (NPsiCTLA-4) were designed and improved both T cell activation and proliferation in vitro. Additionally, the internalization of these NPs by tumor-infiltrating T cells in vivo and CD8+ T cell activation were observed in melanoma-bearing mice [153].

### 4.1. Nano-Immunotherapy Clinical Trials in Cancer

As previously mentioned, preclinical trials have demonstrated that NPs show advantages in improving the efficacy of cancer IT. As a result, different nano-formulations have also been evaluated for cancer treatment in clinical trials. Thus, the following section describes the clinical nano-immunotherapies over the past 5 years (Table 1).

#### 4.1.1. Immune Checkpoint Inhibitors 

The most frequently combinations used in cancer are immune checkpoint blockade antibodies with CT drug NPs. Atezolizumab and durvalumab (anti-PD-L1 antibodies) and pembrolizumab and nivolumab (anti-PD-1 antibodies) are extensively combined with pegylated liposomal doxorubicin and NP-albumin-bound (Nab)-paclitaxel, which is commercially known as Abraxane^®^. 

Due to the therapeutic benefits of atezolizumab as a single agent by providing clinical benefits and a good tolerance in patients with TNBC [154] (NCT01375842), this immune drug has been combined with NPs, which may further improve oncological results. In line with this notion, atezolizumab combined with nab-paclitaxel enhanced the median progression-free survival (mPFS) (7.2 vs. 5.5 months, *p* = 0.002) and the median overall survival (mOS) (21.3 vs. 17.6 months, *p* = 0.08) compared to nab-paclitaxel with placebo in untreated metastatic TNBC (NCT02425891) [155]. Currently, atezolizumab is being tested in combination with nab-paclitaxel in breast and lung cancers. Atezolizumab plus nab-paclitaxel in unresectable locally advanced or metastatic PD-L1-positive TNBC is an active, not recruiting phase III study (NCT04148911), and OS and PFS will be evaluated. In addition, atezolizumab will be combined with carboplatin plus nab-paclitaxel in a recruiting trial (NCT05266937), whose goal is to provide evidence on the efficacy of atezolizumab plus carboplatin plus paclitaxel as a first-line therapy in metastatic PD-L1-positive TNBC. In lung cancer, an exploratory trial intends to evaluate the immune signature to predict the response of atezolizumab plus carboplatin/nab-paclitaxel treatment in patients with resectable stage II, IIIA, and select IIIB (T3N2 only) non-squamous non-small cell lung cancer (NSCLC) (NCT04865250). Moreover, in patients with resectable stage II, IIIa, or select IIIb NSCLC, a phase III multicenter study has been designed to evaluate the efficacy, safety, pharmacokinetics, and immunogenicity of neoadjuvant treatment with atezolizumab (NCT03456063). The combination of atezolizumab plus nab-paclitaxel and carboplatin significantly improved mOS (18.6 vs. 13.9 months, *p* = 0.033) compared with CT in metastatic non-squamous NSCL cancer patients (NCT02367781) [156].

Pembrolizumab has also been combined with different CT drug NPs. In this sense, pembrolizumab and liposomal doxorubicin have been combined in endocrine-resistant BC (NCT03591276), in hormone receptor positive/human epidermal growth factor receptor 2 negative locally recurrent inoperable or metastatic BC(NCT04895358), and also in patients with ovarian cancer (NCT03596281) and their platinum-resistant counterparts (NCT03539328). Additionally, pembrolizumab and nab-paclitaxel is being studied in locally advanced head and neck squamous cell carcinoma (NCT05272696), in patients with locally advanced or metastatic NSCLC (NCT04297605), and in elderly patients with advanced NSCLC (NCT04754815). A completed trial in patients with metastatic urothelial carcinoma (NCT03464734) combining pembrolizumab and nab-paclitaxel showed a favorable safety profile and a mPFS of 5.9 months together with partial and complete responses of 38.6% and 14.3%, respectively ([157], NCT03464734). Finally, a phase II recruiting trial combined pembrolizumab and liposomal irinotecan in TNBC patients with brain metastases (NCT05255666). 

Currently, there are many ongoing studies combining nab-paclitaxel with nivolumab and durvalumab in different types of cancer. For instance, patients with advanced stage NSCLC were treated with a nab-paclitaxel and durvalumab combinatorial treatment (NCT02250326), and the results demonstrated antitumor activity without new safety signals [158]. 

Apart from the clinical trials previously mentioned, another immune checkpoint inhibitor called ipilimumab (a CTLA-4 blocking antibody) is being combined with nivolumab, and with CT drugs encapsulated in NPs, such as nab-paclitaxel or pegylated liposomal doxorubicin, in patients with breast, lung, and pancreatic cancers (NCT03409198, NCT04247165, NCT04132817, NCT04929041).

#### 4.1.2. Other Combinatorial Therapies

Regarding NBTXR3, a phase II/III trial checked the safety and efficacy of NBTXR3 activated by RT in patients with soft tissue sarcoma (NCT02379845). Complete responses were observed in 16% of patients in the NBTXR3 group and 8% in the group of patients treated with RT alone (*p* = 0.044) [159]. However, 39% and 30% of patients had serious adverse events in both groups, respectively [159]. There are currently five clinical trials, and four of them are in the recruiting phase. Thus, NBTXR3 will be evaluated in combination with nivolumab, pembrolizumab, and ipilimumab (NCT03589339, NCT05039632, NCT04484909, NCT04615013, and NCT04862455). These I and II phase trials have the main objective of determining the safety, possible side effects, possible benefits, and the recommended phase II dose of NBTXR3 in combination with other drugs. 

Finally, it should be pointed out that the FDA has already approved the iron oxide NP ferumoxytol due to its effects against tumor cells and TME [144,160]. Only two clinical trials have been conducted over the past 5 years: a recruiting trial in patients with primary and metastatic hepatic cancers combining ferumoxytol with RT (NCT04682847) and a phase I trial where a safety of pharmacologic ascorbate and ferumoxytol with standard of care chemoradiation in glioblastoma patients will be evaluated (NCT04900792).

## 5. Conclusions

Nanotechnology is the latest approach for diagnosis and treatment for cancer, which has represented an alternative in the field of drug administration to treat this disease. This is due to the intrinsic characteristics of nanoparticles used, which provide more specific and efficient drug delivery, decreasing the risk of side effects. Indeed, one of the emerging tracks of nanotechnology is their application in the field of IT. Several preclinical and clinical studies have described how cancer IT has achieved excellent therapeutic effects and has advantages over traditional treatments in many types of tumors. However, IT has obvious limitations, including poor therapeutic targeting, side effects, or TME-promoted immunosuppression, among many others. To this end, researchers are working on different strategies to develop and improve the design and functionality of nanocarriers such as improving the stability of NPs in the bloodstream or by increasing the drug loading capacity. In addition, advances in manufacturing techniques are enabling the creation of NPs with more effective sizes, shapes, and surface chemistry that can influence their behavior within the body.

NP-based cancer IT is still in an early stage of development, but it is clearly a promising strategy that has shown huge preclinical and clinical potential in the last years. In addition, nanotechnology allows us to effectively target both immune and tumor cells, improving cancer IT. In fact, a promising avenue for overcoming the limitations is the design of NPs to transport immune-stimulating molecules, such as checkpoint inhibitors or cytokines, directly to the tumor site, which is being confirmed to minimize unwanted effects and maximize the immune response against tumor cells, leading to improved therapeutic outcomes.

Altogether, this suggests that the combinatorial treatment may induce stronger antitumor immune responses, inhibit the immunosuppression driven by the TME and its components, and directly attack tumor cells, which would greatly improve clinical outcomes. Moreover, from a molecular point of view, our knowledge about the interaction between tumor cells and immune cells, as well as their interaction with nanoparticles, will be enhanced. For this, the development of multifunctional NPs is gaining momentum, allowing not only drug delivery but also the incorporation of additional elements, such as imaging agents or immunostimulatory molecules. This approach has the potential for real-time monitoring of tumor and immune response.

In addition, key biomarkers to develop more specific and multifunctional NPs should be identified, although it is currently a limitation and further research is still needed in this field. These new NPs, therefore, would enhance the efficacy of IT, mostly by targeted delivery. Although the use of NP-based cancer IT has showed attractive potential mainly in preclinical studies, there are still different obstacles. Some of the limitations are the translation to clinical settings, the need for analyzing current and new immune biomarkers, or the rational design of new NPs and combinatorial schedules to personalize cancer treatments according to the types of patients and cancers, which will bring more effective immunotherapeutic strategies and approaches to cancer patients. By combining the precision of targeted drug delivery with the power of immune system activation, NPs offers a promising avenue for overcoming the limitations of conventional therapies and achieving more durable responses.

The development of multi-functional nanoparticles, capable of simultaneously delivering chemotherapeutic agents, immunomodulatory molecules, and imaging probes, holds the key to unlocking the full therapeutic potential of this approach.

## Figures and Tables

**Table 1:** Clinical trials using nanoparticles as immunotherapeutic approaches in cancer.

NCT Number	Clinical Trial Phase	Type of Cancer	Current Status	Nanoparticles	Additional Treatments and Procedures
NCT03589339	1	Bladder Breast Cervix Head and neck Liver Lung Melanoma Renal	Recruiting	NBTXR3	Nivolumab, pembrolizumab, and stereotactic ablative body radiotherapy (SABRT)
NCT05039632	1 and 2	Liver Lung	Not yet recruiting	NBTXR3	Ipilimumab, nivolumab, and abscopal RT
NCT04484909	1	Pancreatic	Recruiting	NBTXR3	RT
NCT04615013	1	Esophageal	Recruiting	NBTXR3	Capecitabine, carboplatin, docetaxel, fluorouracil, leucovorin, oxaliplatin, paclitaxel, and intensity-modulated RT
NCT02379845	2 and 3	Sarcoma	Completed	NBTXR3	RT
NCT04862455	2	Head and neck	Recruiting	NBTXR3	Pembrolizumab, hypofractionated RT, and SBRT
NCT03464734	2	Urothelial	Completed	Nanoparticle albumin-bound (Nab) paclitaxel	Pembrolizumab
NCT04247165	1 and 2	Pancreatic	Recruiting	Nab-paclitaxel	Gemcitabine, nivolumab, ipilimumab, and SBRT
NCT04132817	1	Breast	Active, not recruiting	Nab-paclitaxel	Nivolumab and ipilimumab
NCT04929041	2 and 3	Lung	Recruiting	Nab-paclitaxel	Carboplatin, ipilimumab, nivolumab, pembrolizumab, pemetrexed, and SBRT
NCT04148911	3	Breast	Active, not recruiting	Nab-paclitaxel	Atezolizumab
NCT05266937	2	Breast	Recruiting	Nab-paclitaxel	Atezolizumab and carboplatin
NCT04865250	2	Lung	Recruiting	Nab-paclitaxel	Atezolizumab and carboplatin
NCT03456063	3	Lung	Active, not recruiting	Nab-paclitaxel	Atezolizumab, pemetrexed, carboplatin, cisplatin, and gemcitabine
NCT05272696	2	Head and neck	Not yet recruiting	Nab-paclitaxel	Cisplatin, pembrolizumab, adjuvant chemoradiotherapy, and surgery
NCT04297605	1	Lung	Recruiting	Nab-paclitaxel	Pembrolizumab and pemetrexed
NCT04754815	2	Lung	Withdrawn	Nab-paclitaxel	Pembrolizumab and pemetrexed
NCT02425891	3	Breast	Completed	Nab-paclitaxel	Atezolizumab
NCT02367781	3	Lung	Completed	Nab-paclitaxel	Atezolizumab, carboplatin, and pemetrexed
NCT02250326	2	Lung	Active, not recruiting	Nab-paclitaxel	CC-486 and duravalumab
NCT04895358	3	Breast	Recruiting	Nab-paclitaxel and liposomal doxorubicin	Pembrolizumab, capecitabine, and dextrose
NCT03539328	2	Ovarian	Unknown	Liposomal doxorubicin	Pembrolizumab, gemcitabine, and paclitaxel
NCT03591276	1 and 2	Breast	Recruiting	Pegylated liposomal doxorubicin	Pembrolizumab
NCT05255666	2	Breast	Recruiting	Liposomal Irinotecan	Pembrolizumab
NCT03596281	1	Ovarian	Active, not recruiting	Pegylated liposomal doxorubicin	Pembrolizumab and bevacizumab
NCT03409198	2	Breast	Completed	Pegylated liposomal doxorubicin	Ipilimumab, nivolumab, and cyclophosphamide
NCT04682847	Unknown	Liver	Recruiting	Ferumoxytol	Adaptive Stereotactic RT
NCT04900792	1	Glioblastoma	Not yet recruiting	Ferumoxytol	Ascorbate, temozolomide, and external beam RT

## References

[1] Garcia-Dominguez D.J., Hontecillas-Prieto L., Palazon-Carrion N., Jimenez-Cortegana C., Sanchez-Margalet V., de la Cruz-Merino L. (2022). Tumor Immune Microenvironment in Lymphoma: Focus on Epigenetics. Cancers.

[2] Baylin S.B., Jones P.A. (2011). A decade of exploring the cancer epigenome—Biological and translational implications. Nat. Rev. Cancer.

[3] Sandoval J., Esteller M. (2012). Cancer epigenomics: Beyond genomics. Curr. Opin. Genet. Dev..

[4] Arruebo M., Vilaboa N., Saez-Gutierrez B., Lambea J., Tres A., Valladares M., Gonzalez-Fernandez A. (2011). Assessment of the evolution of cancer treatment therapies. Cancers.

[5] Beil D.R., Wein L.M. (2002). Sequencing surgery, radiotherapy and chemotherapy: Insights from a mathematical analysis. Breast Cancer Res. Treat..

[6] Zhang Z., Liu X., Chen D., Yu J. (2022). Radiotherapy combined with immunotherapy: The dawn of cancer treatment. Signal Transduct. Target. Ther..

[7] Pucci C., Martinelli C., Ciofani G. (2019). Innovative approaches for cancer treatment: Current perspectives and new challenges. Ecancermedicalscience.

[8] Urruticoechea A., Alemany R., Balart J., Villanueva A., Vinals F., Capella G. (2010). Recent advances in cancer therapy: An overview. Curr. Pharm. Des..

[9] Cao J., Huang D., Peppas N.A. (2020). Advanced engineered nanoparticulate platforms to address key biological barriers for delivering chemotherapeutic agents to target sites. Adv. Drug Deliv. Rev..

[10] Jiang W., Wang Y., Wargo J.A., Lang F.F., Kim B.Y.S. (2021). Considerations for designing preclinical cancer immune nanomedicine studies. Nat. Nanotechnol..

[11] Waldmann T.A. (2018). Cytokines in Cancer Immunotherapy. Cold Spring Harb. Perspect. Biol..

[12] Chen Z., Wang Z., Gu Z. (2019). Bioinspired and Biomimetic Nanomedicines. Acc. Chem. Res..

[13] Abbas A.K., Lichtman A.H., Pillai S. (2022). Cellular and Molecular Immunology.

[14] Smyth M.J., Dunn G.P., Schreiber R.D. (2006). Cancer immunosurveillance and immunoediting: The roles of immunity in suppressing tumor development and shaping tumor immunogenicity. Adv. Immunol..

[15] Taefehshokr N., Baradaran B., Baghbanzadeh A., Taefehshokr S. (2020). Promising approaches in cancer immunotherapy. Immunobiology.

[16] Tan S., Li D., Zhu X. (2020). Cancer immunotherapy: Pros, cons and beyond. Biomed. Pharmacother..

[17] Rosenberg S.A., Packard B.S., Aebersold P.M., Solomon D., Topalian S.L., Toy S.T., Simon P., Lotze M.T., Yang J.C., Seipp C.A. (1988). Use of tumor-infiltrating lymphocytes and interleukin-2 in the immunotherapy of patients with metastatic melanoma. A preliminary report. N. Engl. J. Med..

[18] Wu R., Forget M.A., Chacon J., Bernatchez C., Haymaker C., Chen J.Q., Hwu P., Radvanyi L.G. (2012). Adoptive T-cell therapy using autologous tumor-infiltrating lymphocytes for metastatic melanoma: Current status and future outlook. Cancer J..

[19] Andersen R., Donia M., Ellebaek E., Borch T.H., Kongsted P., Iversen T.Z., Holmich L.R., Hendel H.W., Met O., Andersen M.H. (2016). Long-Lasting Complete Responses in Patients with Metastatic Melanoma after Adoptive Cell Therapy with Tumor-Infiltrating Lymphocytes and an Attenuated IL2 Regimen. Clin. Cancer Res..

[20] Mehta G.U., Malekzadeh P., Shelton T., White D.E., Butman J.A., Yang J.C., Kammula U.S., Goff S.L., Rosenberg S.A., Sherry R.M. (2018). Outcomes of Adoptive Cell Transfer with Tumor-infiltrating Lymphocytes for Metastatic Melanoma Patients with and without Brain Metastases. J. Immunother..

[21] Hamoud B.H., Sima R.M., Vacaroiu I.A., Georgescu M.T., Bobirca A., Gaube A., Bobirca F., Georgescu D.E. (2023). The Evolving Landscape of Immunotherapy in Uterine Cancer: A Comprehensive Review. Life.

[22] Whiteside T.L. (2008). The tumor microenvironment and its role in promoting tumor growth. Oncogene.

[23] Dudley M.E., Wunderlich J.R., Shelton T.E., Even J., Rosenberg S.A. (2003). Generation of tumor-infiltrating lymphocyte cultures for use in adoptive transfer therapy for melanoma patients. J. Immunother..

[24] Chacon J.A., Wu R.C., Sukhumalchandra P., Molldrem J.J., Sarnaik A., Pilon-Thomas S., Weber J., Hwu P., Radvanyi L. (2013). Co-stimulation through 4-1BB/CD137 improves the expansion and function of CD8(+) melanoma tumor-infiltrating lymphocytes for adoptive T-cell therapy. PLoS ONE.

[25] Ye Q., Song D.G., Poussin M., Yamamoto T., Best A., Li C., Coukos G., Powell D.J. (2014). CD137 accurately identifies and enriches for naturally occurring tumor-reactive T cells in tumor. Clin. Cancer Res..

[26] Khammari A., Knol A.C., Nguyen J.M., Bossard C., Denis M.G., Pandolfino M.C., Quereux G., Bercegeay S., Dreno B. (2014). Adoptive TIL transfer in the adjuvant setting for melanoma: Long-term patient survival. J. Immunol. Res..

[27] Abbas A.K. (2020). The Surprising Story of IL-2: From Experimental Models to Clinical Application. Am. J. Pathol..

[28] Georgescu M.T., Trifanescu O.G., Serbanescu G.L., Mitrica R.I., Georgescu D.E., Mihaila R.I., Neagu A., Gaube A., Botezatu C., Mastalier B.S.M. (2023). Navigating a Complex Intersection: Immunotherapy and Radiotherapy Synergy in Squamous Cell Carcinoma of the Skin—A Comprehensive Literature Review. Cosmetics.

[29] Deniger D.C., Kwong M.L., Pasetto A., Dudley M.E., Wunderlich J.R., Langhan M.M., Lee C.R., Rosenberg S.A. (2017). A Pilot Trial of the Combination of Vemurafenib with Adoptive Cell Therapy in Patients with Metastatic Melanoma. Clin. Cancer Res..

[30] Tarhini A.A., Edington H., Butterfield L.H., Lin Y., Shuai Y., Tawbi H., Sander C., Yin Y., Holtzman M., Johnson J. (2014). Immune monitoring of the circulation and the tumor microenvironment in patients with regionally advanced melanoma receiving neoadjuvant ipilimumab. PLoS ONE.

[31] Frederick D.T., Piris A., Cogdill A.P., Cooper Z.A., Lezcano C., Ferrone C.R., Mitra D., Boni A., Newton L.P., Liu C. (2013). BRAF inhibition is associated with enhanced melanoma antigen expression and a more favorable tumor microenvironment in patients with metastatic melanoma. Clin. Cancer Res..

[32] Vella L.J., Pasam A., Dimopoulos N., Andrews M., Knights A., Puaux A.L., Louahed J., Chen W., Woods K., Cebon J.S. (2014). MEK inhibition, alone or in combination with BRAF inhibition, affects multiple functions of isolated normal human lymphocytes and dendritic cells. Cancer Immunol. Res..

[33] Nagasaki J., Inozume T., Sax N., Ariyasu R., Ishikawa M., Yamashita K., Kawazu M., Ueno T., Irie T., Tanji E. (2022). PD-1 blockade therapy promotes infiltration of tumor-attacking exhausted T cell clonotypes. Cell Rep..

[34] Chan C.Y., Chiu D.K., Yuen V.W., Law C.T., Wong B.P., Thu K.L., Cescon D.W., Soria-Bretones I., Cheu J.W., Lee D. (2022). CFI-402257, a TTK inhibitor, effectively suppresses hepatocellular carcinoma. Proc. Natl. Acad. Sci. USA.

[35] Zacharakis N., Huq L.M., Seitter S.J., Kim S.P., Gartner J.J., Sindiri S., Hill V.K., Li Y.F., Paria B.C., Ray S. (2022). Breast Cancers Are Immunogenic: Immunologic Analyses and a Phase II Pilot Clinical Trial Using Mutation-Reactive Autologous Lymphocytes. J. Clin. Oncol..

[36] Creelan B.C., Wang C., Teer J.K., Toloza E.M., Yao J., Kim S., Landin A.M., Mullinax J.E., Saller J.J., Saltos A.N. (2021). Tumor-infiltrating lymphocyte treatment for anti-PD-1-resistant metastatic lung cancer: A phase 1 trial. Nat. Med..

[37] Yee C. (2014). The use of endogenous T cells for adoptive transfer. Immunol. Rev..

[38] Shao H., Ou Y., Wang T., Shen H., Wu F., Zhang W., Tao C., Yuan Y., Bo H., Wang H. (2014). Differences in TCR-Vbeta repertoire and effector phenotype between tumor infiltrating lymphocytes and peripheral blood lymphocytes increase with age. PLoS ONE.

[39] Yee C. (2006). Adoptive T-cell therapy of cancer. Hematol. Oncol. Clin. N. Am..

[40] Chapuis A.G., Lee S.M., Thompson J.A., Roberts I.M., Margolin K.A., Bhatia S., Sloan H.L., Lai I., Wagener F., Shibuya K. (2016). Combined IL-21-primed polyclonal CTL plus CTLA4 blockade controls refractory metastatic melanoma in a patient. J. Exp. Med..

[41] Turtle C.J., Hanafi L.A., Berger C., Gooley T.A., Cherian S., Hudecek M., Sommermeyer D., Melville K., Pender B., Budiarto T.M. (2016). CD19 CAR-T cells of defined CD4+:CD8+ composition in adult B cell ALL patients. J. Clin. Investig..

[42] Davila M.L., Brentjens R.J. (2016). CD19-Targeted CAR T cells as novel cancer immunotherapy for relapsed or refractory B-cell acute lymphoblastic leukemia. Clin. Adv. Hematol. Oncol..

[43] Kwok A., Eggimann G.A., Reymond J.L., Darbre T., Hollfelder F. (2013). Peptide dendrimer/lipid hybrid systems are efficient DNA transfection reagents: Structure–activity relationships highlight the role of charge distribution across dendrimer generations. ACS Nano.

[44] Liao X., Li Y., Bonini C., Nair S., Gilboa E., Greenberg P.D., Yee C. (2004). Transfection of RNA encoding tumor antigens following maturation of dendritic cells leads to prolonged presentation of antigen and the generation of high-affinity tumor-reactive cytotoxic T lymphocytes. Mol. Ther..

[45] Chen Y.Z., Yao X.L., Tabata Y., Nakagawa S., Gao J.Q. (2010). Gene carriers and transfection systems used in the recombination of dendritic cells for effective cancer immunotherapy. Clin. Dev. Immunol..

[46] Butler M.O., Lee J.S., Ansen S., Neuberg D., Hodi F.S., Murray A.P., Drury L., Berezovskaya A., Mulligan R.C., Nadler L.M. (2007). Long-lived antitumor CD8+ lymphocytes for adoptive therapy generated using an artificial antigen-presenting cell. Clin. Cancer Res..

[47] Chapuis A.G., Thompson J.A., Margolin K.A., Rodmyre R., Lai I.P., Dowdy K., Farrar E.A., Bhatia S., Sabath D.E., Cao J. (2012). Transferred melanoma-specific CD8+ T cells persist, mediate tumor regression, and acquire central memory phenotype. Proc. Natl. Acad. Sci. USA.

[48] Chapuis A.G., Desmarais C., Emerson R., Schmitt T.M., Shibuya K., Lai I., Wagener F., Chou J., Roberts I.M., Coffey D.G. (2017). Tracking the Fate and Origin of Clinically Relevant Adoptively Transferred CD8(+) T Cells In Vivo. Sci. Immunol..

[49] Li Y., Bleakley M., Yee C. (2005). IL-21 influences the frequency, phenotype, and affinity of the antigen-specific CD8 T cell response. J. Immunol..

[50] Li Y., Yee C. (2008). IL-21 mediated Foxp3 suppression leads to enhanced generation of antigen-specific CD8+ cytotoxic T lymphocytes. Blood.

[51] Pollack S.M., Jones R.L., Farrar E.A., Lai I.P., Lee S.M., Cao J., Pillarisetty V.G., Hoch B.L., Gullett A., Bleakley M. (2014). Tetramer guided, cell sorter assisted production of clinical grade autologous NY-ESO-1 specific CD8(+) T cells. J. Immunother. Cancer.

[52] Ishihara M., Kitano S., Kageyama S., Miyahara Y., Yamamoto N., Kato H., Mishima H., Hattori H., Funakoshi T., Kojima T. (2022). NY-ESO-1-specific redirected T cells with endogenous TCR knockdown mediate tumor response and cytokine release syndrome. J. Immunother. Cancer.

[53] Roth T.L., Puig-Saus C., Yu R., Shifrut E., Carnevale J., Li P.J., Hiatt J., Saco J., Krystofinski P., Li H. (2018). Reprogramming human T cell function and specificity with non-viral genome targeting. Nature.

[54] Kebriaei P., Singh H., Huls M.H., Figliola M.J., Bassett R., Olivares S., Jena B., Dawson M.J., Kumaresan P.R., Su S. (2016). Phase I trials using Sleeping Beauty to generate CD19-specific CAR T cells. J. Clin. Investig..

[55] Beatty G.L., Haas A.R., Maus M.V., Torigian D.A., Soulen M.C., Plesa G., Chew A., Zhao Y., Levine B.L., Albelda S.M. (2014). Mesothelin-specific chimeric antigen receptor mRNA-engineered T cells induce anti-tumor activity in solid malignancies. Cancer Immunol. Res..

[56] Amir A.L., van der Steen D.M., van Loenen M.M., Hagedoorn R.S., de Boer R., Kester M.D., de Ru A.H., Lugthart G.J., van Kooten C., Hiemstra P.S. (2011). PRAME-specific Allo-HLA-restricted T cells with potent antitumor reactivity useful for therapeutic T-cell receptor gene transfer. Clin. Cancer Res..

[57] Zhao Y., Bennett A.D., Zheng Z., Wang Q.J., Robbins P.F., Yu L.Y., Li Y., Molloy P.E., Dunn S.M., Jakobsen B.K. (2007). High-affinity TCRs generated by phage display provide CD4+ T cells with the ability to recognize and kill tumor cell lines. J. Immunol..

[58] Morgan R.A., Dudley M.E., Wunderlich J.R., Hughes M.S., Yang J.C., Sherry R.M., Royal R.E., Topalian S.L., Kammula U.S., Restifo N.P. (2006). Cancer regression in patients after transfer of genetically engineered lymphocytes. Science.

[59] Robbins P.F., Kassim S.H., Tran T.L., Crystal J.S., Morgan R.A., Feldman S.A., Yang J.C., Dudley M.E., Wunderlich J.R., Sherry R.M. (2015). A pilot trial using lymphocytes genetically engineered with an NY-ESO-1-reactive T-cell receptor: Long-term follow-up and correlates with response. Clin. Cancer Res..

[60] Robbins P.F., Morgan R.A., Feldman S.A., Yang J.C., Sherry R.M., Dudley M.E., Wunderlich J.R., Nahvi A.V., Helman L.J., Mackall C.L. (2011). Tumor regression in patients with metastatic synovial cell sarcoma and melanoma using genetically engineered lymphocytes reactive with NY-ESO-1. J. Clin. Oncol..

[61] Rapoport A.P., Stadtmauer E.A., Binder-Scholl G.K., Goloubeva O., Vogl D.T., Lacey S.F., Badros A.Z., Garfall A., Weiss B., Finklestein J. (2015). NY-ESO-1-specific TCR-engineered T cells mediate sustained antigen-specific antitumor effects in myeloma. Nat. Med..

[62] Morgan R.A., Chinnasamy N., Abate-Daga D., Gros A., Robbins P.F., Zheng Z., Dudley M.E., Feldman S.A., Yang J.C., Sherry R.M. (2013). Cancer regression and neurological toxicity following anti-MAGE-A3 TCR gene therapy. J. Immunother..

[63] Cameron B.J., Gerry A.B., Dukes J., Harper J.V., Kannan V., Bianchi F.C., Grand F., Brewer J.E., Gupta M., Plesa G. (2013). Identification of a Titin-derived HLA-A1-presented peptide as a cross-reactive target for engineered MAGE A3-directed T cells. Sci. Transl. Med..

[64] Linette G.P., Stadtmauer E.A., Maus M.V., Rapoport A.P., Levine B.L., Emery L., Litzky L., Bagg A., Carreno B.M., Cimino P.J. (2013). Cardiovascular toxicity and titin cross-reactivity of affinity-enhanced T cells in myeloma and melanoma. Blood.

[65] Chambers C.A., Allison J.P. (1997). Co-stimulation in T cell responses. Curr. Opin. Immunol..

[66] Hombach A.A., Heiders J., Foppe M., Chmielewski M., Abken H. (2012). OX40 costimulation by a chimeric antigen receptor abrogates CD28 and IL-2 induced IL-10 secretion by redirected CD4(+) T cells. Oncoimmunology.

[67] Jaspers J.E., Brentjens R.J. (2017). Development of CAR T cells designed to improve antitumor efficacy and safety. Pharmacol. Ther..

[68] Thanindratarn P., Dean D.C., Nelson S.D., Hornicek F.J., Duan Z. (2020). Chimeric antigen receptor T (CAR-T) cell immunotherapy for sarcomas: From mechanisms to potential clinical applications. Cancer Treat. Rev..

[69] Fathi Maroufi N., Aghayi E., Garshasbi H., Gholampour Matin M., Babazadeh Bedoustani A., Firouzi Amoudizaj F., Hajazimian S., Isazadeh A., Taefehshokr S., Taefehshokr N. (2019). Association of rs1946518 C/A Polymorphism in Promoter Region of Interleukin 18 Gene and Breast Cancer Risk in Iranian Women: A Case-control Study. Iran. J. Allergy Asthma Immunol..

[70] Posey A.D., Schwab R.D., Boesteanu A.C., Steentoft C., Mandel U., Engels B., Stone J.D., Madsen T.D., Schreiber K., Haines K.M. (2016). Engineered CAR T Cells Targeting the Cancer-Associated Tn-Glycoform of the Membrane Mucin MUC1 Control Adenocarcinoma. Immunity.

[71] Vairy S., Garcia J.L., Teira P., Bittencourt H. (2018). CTL019 (tisagenlecleucel): CAR-T therapy for relapsed and refractory B-cell acute lymphoblastic leukemia. Drug Des. Devel Ther..

[72] Schuster S.J., Bishop M.R., Tam C.S., Waller E.K., Borchmann P., McGuirk J.P., Jager U., Jaglowski S., Andreadis C., Westin J.R. (2019). Tisagenlecleucel in Adult Relapsed or Refractory Diffuse Large B-Cell Lymphoma. N. Engl. J. Med..

[73] Geyer M.B. (2019). First CAR to Pass the Road Test: Tisagenlecleucel’s Drive to FDA Approval. Clin. Cancer Res..

[74] Baumeister S.H., Murad J., Werner L., Daley H., Trebeden-Negre H., Gicobi J.K., Schmucker A., Reder J., Sentman C.L., Gilham D.E. (2019). Phase I Trial of Autologous CAR T Cells Targeting NKG2D Ligands in Patients with AML/MDS and Multiple Myeloma. Cancer Immunol. Res..

[75] Sallman D.A., Brayer J., Sagatys E.M., Lonez C., Breman E., Agaugue S., Verma B., Gilham D.E., Lehmann F.F., Davila M.L. (2018). NKG2D-based chimeric antigen receptor therapy induced remission in a relapsed/refractory acute myeloid leukemia patient. Haematologica.

[76] Zhang H., Gan W.T., Hao W.G., Wang P.F., Li Z.Y., Chang L.J. (2020). Successful Anti-CLL1 CAR T-Cell Therapy in Secondary Acute Myeloid Leukemia. Front. Oncol..

[77] Jetani H., Garcia-Cadenas I., Nerreter T., Thomas S., Rydzek J., Meijide J.B., Bonig H., Herr W., Sierra J., Einsele H. (2018). CAR T-cells targeting FLT3 have potent activity against FLT3(−)ITD(+) AML and act synergistically with the FLT3-inhibitor crenolanib. Leukemia.

[78] Wang Y., Xu Y., Li S., Liu J., Xing Y., Xing H., Tian Z., Tang K., Rao Q., Wang M. (2018). Targeting FLT3 in acute myeloid leukemia using ligand-based chimeric antigen receptor-engineered T cells. J. Hematol. Oncol..

[79] Sommer C., Cheng H.Y., Nguyen D., Dettling D., Yeung Y.A., Sutton J., Hamze M., Valton J., Smith J., Djuretic I. (2020). Allogeneic FLT3 CAR T Cells with an Off-Switch Exhibit Potent Activity against AML and Can Be Depleted to Expedite Bone Marrow Recovery. Mol. Ther..

[80] Marin V., Pizzitola I., Agostoni V., Attianese G.M., Finney H., Lawson A., Pule M., Rousseau R., Biondi A., Biagi E. (2010). Cytokine-induced killer cells for cell therapy of acute myeloid leukemia: Improvement of their immune activity by expression of CD33-specific chimeric receptors. Haematologica.

[81] Dutour A., Marin V., Pizzitola I., Valsesia-Wittmann S., Lee D., Yvon E., Finney H., Lawson A., Brenner M., Biondi A. (2012). In Vitro and In Vivo Antitumor Effect of Anti-CD33 Chimeric Receptor-Expressing EBV-CTL against CD33 Acute Myeloid Leukemia. Adv. Hematol..

[82] Economides M.P., McCue D., Lane A.A., Pemmaraju N. (2019). Tagraxofusp, the first CD123-targeted therapy and first targeted treatment for blastic plasmacytoid dendritic cell neoplasm. Expert. Rev. Clin. Pharmacol..

[83] Uy G.L., Aldoss I., Foster M.C., Sayre P.H., Wieduwilt M.J., Advani A.S., Godwin J.E., Arellano M.L., Sweet K.L., Emadi A. (2021). Flotetuzumab as salvage immunotherapy for refractory acute myeloid leukemia. Blood.

[84] Morris E.C., Neelapu S.S., Giavridis T., Sadelain M. (2022). Cytokine release syndrome and associated neurotoxicity in cancer immunotherapy. Nat. Rev. Immunol..

[85] Neelapu S.S., Locke F.L., Bartlett N.L., Lekakis L.J., Miklos D.B., Jacobson C.A., Braunschweig I., Oluwole O.O., Siddiqi T., Lin Y. (2017). Axicabtagene Ciloleucel CAR T-Cell Therapy in Refractory Large B-Cell Lymphoma. N. Engl. J. Med..

[86] Slaney C.Y., Kershaw M.H., Darcy P.K. (2014). Trafficking of T cells into tumors. Cancer Res..

[87] Craddock J.A., Lu A., Bear A., Pule M., Brenner M.K., Rooney C.M., Foster A.E. (2010). Enhanced tumor trafficking of GD2 chimeric antigen receptor T cells by expression of the chemokine receptor CCR2b. J. Immunother..

[88] Giuffrida L., Sek K., Henderson M.A., House I.G., Lai J., Chen A.X.Y., Todd K.L., Petley E.V., Mardiana S., Todorovski I. (2020). IL-15 Preconditioning Augments CAR T Cell Responses to Checkpoint Blockade for Improved Treatment of Solid Tumors. Mol. Ther..

[89] Song Y., Gao Q., Zhang H., Fan L., Zhou J., Zou D., Li W., Yang H., Liu T., Wang Q. (2020). Treatment of relapsed or refractory classical Hodgkin lymphoma with the anti-PD-1, tislelizumab: Results of a phase 2, single-arm, multicenter study. Leukemia.

[90] Guedan S., Madar A., Casado-Medrano V., Shaw C., Wing A., Liu F., Young R.M., June C.H., Posey A.D. (2020). Single residue in CD28-costimulated CAR-T cells limits long-term persistence and antitumor durability. J. Clin. Investig..

[91] Ghosn M., Cheema W., Zhu A., Livschitz J., Maybody M., Boas F.E., Santos E., Kim D., Beattie J.A., Offin M. (2022). Image-guided interventional radiological delivery of chimeric antigen receptor (CAR) T cells for pleural malignancies in a phase I/II clinical trial. Lung Cancer.

[92] Adusumilli P.S., Zauderer M.G., Riviere I., Solomon S.B., Rusch V.W., O’Cearbhaill R.E., Zhu A., Cheema W., Chintala N.K., Halton E. (2021). A Phase I Trial of Regional Mesothelin-Targeted CAR T-cell Therapy in Patients with Malignant Pleural Disease, in Combination with the Anti-PD-1 Agent Pembrolizumab. Cancer Discov..

[93] Chen L., Chen F., Niu H., Li J., Pu Y., Yang C., Wang Y., Huang R., Li K., Lei Y. (2022). Chimeric Antigen Receptor (CAR)-T Cell Immunotherapy against Thoracic Malignancies: Challenges and Opportunities. Front. Immunol..

[94] Tinkle S., McNeil S.E., Muhlebach S., Bawa R., Borchard G., Barenholz Y.C., Tamarkin L., Desai N. (2014). Nanomedicines: Addressing the scientific and regulatory gap. Ann. N. Y. Acad. Sci..

[95] Cheng Z., Li M., Dey R., Chen Y. (2021). Nanomaterials for cancer therapy: Current progress and perspectives. J. Hematol. Oncol..

[96] Heshmati Aghda N., Dabbaghianamiri M., Tunnell J.W., Betancourt T. (2022). Design of smart nanomedicines for effective cancer treatment. Int. J. Pharm..

[97] Sriraman S.K., Aryasomayajula B., Torchilin V.P. (2014). Barriers to drug delivery in solid tumors. Tissue Barriers.

[98] Garnacho C. (2016). Intracellular Drug Delivery: Mechanisms for Cell Entry. Curr. Pharm. Des..

[99] Mitchell M.J., Billingsley M.M., Haley R.M., Wechsler M.E., Peppas N.A., Langer R. (2021). Engineering precision nanoparticles for drug delivery. Nat. Rev. Drug Discov..

[100] Meir R., Shamalov K., Betzer O., Motiei M., Horovitz-Fried M., Yehuda R., Popovtzer A., Popovtzer R., Cohen C.J. (2015). Nanomedicine for Cancer Immunotherapy: Tracking Cancer-Specific T-Cells in Vivo with Gold Nanoparticles and CT Imaging. ACS Nano.

[101] Chenthamara D., Subramaniam S., Ramakrishnan S.G., Krishnaswamy S., Essa M.M., Lin F.H., Qoronfleh M.W. (2019). Therapeutic efficacy of nanoparticles and routes of administration. Biomater. Res..

[102] Haist M., Mailander V., Bros M. (2022). Nanodrugs Targeting T Cells in Tumor Therapy. Front. Immunol..

[103] Alizadeh D., Trad M., Hanke N.T., Larmonier C.B., Janikashvili N., Bonnotte B., Katsanis E., Larmonier N. (2014). Doxorubicin eliminates myeloid-derived suppressor cells and enhances the efficacy of adoptive T-cell transfer in breast cancer. Cancer Res..

[104] Din F.U., Aman W., Ullah I., Qureshi O.S., Mustapha O., Shafique S., Zeb A. (2017). Effective use of nanocarriers as drug delivery systems for the treatment of selected tumors. Int. J. Nanomed..

[105] Adriano B., Cotto N.M., Chauhan N., Jaggi M., Chauhan S.C., Yallapu M.M. (2021). Milk exosomes: Nature’s abundant nanoplatform for theranostic applications. Bioact. Mater..

[106] Ding X., Liu D., Booth G., Gao W., Lu Y. (2018). Virus-Like Particle Engineering: From Rational Design to Versatile Applications. Biotechnol. J..

[107] Yang Y., Chen Y., Zhang F., Zhao Q., Zhong H. (2015). Increased anti-tumour activity by exosomes derived from doxorubicin-treated tumour cells via heat stress. Int. J. Hyperth..

[108] Hadla M., Palazzolo S., Corona G., Caligiuri I., Canzonieri V., Toffoli G., Rizzolio F. (2016). Exosomes increase the therapeutic index of doxorubicin in breast and ovarian cancer mouse models. Nanomedicine.

[109] Gehrmann U., Hiltbrunner S., Georgoudaki A.M., Karlsson M.C., Naslund T.I., Gabrielsson S. (2013). Synergistic induction of adaptive antitumor immunity by codelivery of antigen with alpha-galactosylceramide on exosomes. Cancer Res..

[110] Rosenblum D., Joshi N., Tao W., Karp J.M., Peer D. (2018). Progress and challenges towards targeted delivery of cancer therapeutics. Nat. Commun..

[111] Blanco E., Shen H., Ferrari M. (2015). Principles of nanoparticle design for overcoming biological barriers to drug delivery. Nat. Biotechnol..

[112] Matsumura Y., Maeda H. (1986). A new concept for macromolecular therapeutics in cancer chemotherapy: Mechanism of tumoritropic accumulation of proteins and the antitumor agent smancs. Cancer Res..

[113] Maeda H., Wu J., Sawa T., Matsumura Y., Hori K. (2000). Tumor vascular permeability and the EPR effect in macromolecular therapeutics: A review. J. Control. Release Off. J. Control. Release Soc..

[114] Zi Y., Yang K., He J., Wu Z., Liu J., Zhang W. (2022). Strategies to enhance drug delivery to solid tumors by harnessing the EPR effects and alternative targeting mechanisms. Adv. Drug Deliv. Rev..

[115] Neri D., Bicknell R. (2005). Tumour vascular targeting. Nat. Rev. Cancer.

[116] Liao J., Zheng H., Fei Z., Lu B., Li D., Xiong X., Yi Y. (2018). Tumor-targeting and pH-responsive nanoparticles from hyaluronic acid for the enhanced delivery of doxorubicin. Int. J. Biol. Macromol..

[117] Liao J., Zheng H., Hu R., Cao J., Wei X., Li D., Yin Y. (2018). Hyaluronan Based Tumor-Targeting and pH-Responsive Shell Cross-Linkable Nanoparticles for the Controlled Release of Doxorubicin. J. Biomed. Nanotechnol..

[118] Bansal K.K., Özliseli E., Rosling A., Rosenholm J.M. (2021). Synthesis and Evaluation of Novel Functional Polymers Derived from Renewable Jasmine Lactone for StimuliResponsive Drug Delivery. Adv. Funct. Mater..

[119] Tian M., Xin X., Wu R., Guan W., Zhou W. (2022). Advances in intelligent-responsive nanocarriers for cancer therapy. Pharmacol. Res..

[120] Graff B.A., Vangberg L., Rofstad E.K. (2004). Quantitative assessment of uptake and distribution of iron oxide particles (NC100150) in human melanoma xenografts by contrast-enhanced MRI. Magn. Reson. Med..

[121] Pun S.H., Tack F., Bellocq N.C., Cheng J., Grubbs B.H., Jensen G.S., Davis M.E., Brewster M., Janicot M., Janssens B. (2004). Targeted delivery of RNA-cleaving DNA enzyme (DNAzyme) to tumor tissue by transferrin-modified, cyclodextrin-based particles. Cancer Biol. Ther..

[122] Kuhn S.J., Finch S.K., Hallahan D.E., Giorgio T.D. (2006). Proteolytic surface functionalization enhances in vitro magnetic nanoparticle mobility through extracellular matrix. Nano Lett..

[123] Goodman T.T., Olive P.L., Pun S.H. (2007). Increased nanoparticle penetration in collagenase-treated multicellular spheroids. Int. J. Nanomed..

[124] Dietz H.C. (2010). TGF-beta in the pathogenesis and prevention of disease: A matter of aneurysmic proportions. J. Clin. Investig..

[125] Diop-Frimpong B., Chauhan V.P., Krane S., Boucher Y., Jain R.K. (2011). Losartan inhibits collagen I synthesis and improves the distribution and efficacy of nanotherapeutics in tumors. Proc. Natl. Acad. Sci. USA.

[126] Shan X., Gong X., Li J., Wen J., Li Y., Zhang Z. (2022). Current approaches of nanomedicines in the market and various stage of clinical translation. Acta Pharm. Sin. B.

[127] Moon J.J., Huang B., Irvine D.J. (2012). Engineering nano- and microparticles to tune immunity. Adv. Mater..

[128] Riley R.S., June C.H., Langer R., Mitchell M.J. (2019). Delivery technologies for cancer immunotherapy. Nat. Rev. Drug Discov..

[129] Duan X., Chan C., Lin W. (2019). Nanoparticle-Mediated Immunogenic Cell Death Enables and Potentiates Cancer Immunotherapy. Angew. Chem. Int. Ed. Engl..

[130] Galluzzi L., Buque A., Kepp O., Zitvogel L., Kroemer G. (2017). Immunogenic cell death in cancer and infectious disease. Nat. Rev. Immunol..

[131] Zhao X., Yang K., Zhao R., Ji T., Wang X., Yang X., Zhang Y., Cheng K., Liu S., Hao J. (2016). Inducing enhanced immunogenic cell death with nanocarrier-based drug delivery systems for pancreatic cancer therapy. Biomaterials.

[132] Chen Q., Liu L., Lu Y., Chen X., Zhang Y., Zhou W., Guo Q., Li C., Zhang Y., Zhang Y. (2019). Tumor Microenvironment-Triggered Aggregated Magnetic Nanoparticles for Reinforced Image-Guided Immunogenic Chemotherapy. Adv. Sci..

[133] Song W., Shen L., Wang Y., Liu Q., Goodwin T.J., Li J., Dorosheva O., Liu T., Liu R., Huang L. (2018). Synergistic and low adverse effect cancer immunotherapy by immunogenic chemotherapy and locally expressed PD-L1 trap. Nat. Commun..

[134] Tang W., Yang J., Yuan Y., Zhao Z., Lian Z., Liang G. (2017). Paclitaxel nanoparticle awakens immune system to fight against cancer. Nanoscale.

[135] Roy A., Chandra S., Mamilapally S., Upadhyay P., Bhaskar S. (2012). Anticancer and immunostimulatory activity by conjugate of paclitaxel and non-toxic derivative of LPS for combined chemo-immunotherapy. Pharm. Res..

[136] Zheng D.W., Chen J.L., Zhu J.Y., Rong L., Li B., Lei Q., Fan J.X., Zou M.Z., Li C., Cheng S.X. (2016). Highly Integrated Nano-Platform for Breaking the Barrier between Chemotherapy and Immunotherapy. Nano Lett..

[137] Gao F., Zhang C., Qiu W.X., Dong X., Zheng D.W., Wu W., Zhang X.Z. (2018). PD-1 Blockade for Improving the Antitumor Efficiency of Polymer-Doxorubicin Nanoprodrug. Small.

[138] Wu J., Tang C., Yin C. (2017). Co-delivery of doxorubicin and interleukin-2 via chitosan based nanoparticles for enhanced antitumor efficacy. Acta Biomater..

[139] Hu Y., Paris S., Barsoumian H., Abana C.O., He K., Wasley M., Younes A.I., Masrorpour F., Chen D., Yang L. (2021). Radiation Therapy Enhanced by NBTXR3 Nanoparticles Overcomes Anti-PD1 Resistance and Evokes Abscopal Effects. Int. J. Radiat. Oncol. Biol. Phys..

[140] Chen Q., Chen J., Yang Z., Xu J., Xu L., Liang C., Han X., Liu Z. (2019). Nanoparticle-Enhanced Radiotherapy to Trigger Robust Cancer Immunotherapy. Adv. Mater..

[141] Cano-Mejia J., Shukla A., Ledezma D.K., Palmer E., Villagra A., Fernandes R. (2020). CpG-coated prussian blue nanoparticles-based photothermal therapy combined with anti-CTLA-4 immune checkpoint blockade triggers a robust abscopal effect against neuroblastoma. Transl. Oncol..

[142] Zhou B., Wu Q., Wang M., Hoover A., Wang X., Zhou F., Towner R.A., Smith N., Saunders D., Song J. (2020). Immunologically modified MnFe_2_O_4_ nanoparticles to synergize photothermal therapy and immunotherapy for cancer treatment. Chem. Eng. J..

[143] Sacchetti C., Rapini N., Magrini A., Cirelli E., Bellucci S., Mattei M., Rosato N., Bottini N., Bottini M. (2013). In vivo targeting of intratumor regulatory T cells using PEG-modified single-walled carbon nanotubes. Bioconjug Chem..

[144] Zanganeh S., Hutter G., Spitler R., Lenkov O., Mahmoudi M., Shaw A., Pajarinen J.S., Nejadnik H., Goodman S., Moseley M. (2016). Iron oxide nanoparticles inhibit tumour growth by inducing pro-inflammatory macrophage polarization in tumour tissues. Nat. Nanotechnol..

[145] Zhang Y., Wu L., Li Z., Zhang W., Luo F., Chu Y., Chen G. (2018). Glycocalyx-Mimicking Nanoparticles Improve Anti-PD-L1 Cancer Immunotherapy through Reversion of Tumor-Associated Macrophages. Biomacromolecules.

[146] He W., Liang P., Guo G., Huang Z., Niu Y., Dong L., Wang C., Zhang J. (2016). Re-polarizing Myeloid-derived Suppressor Cells (MDSCs) with Cationic Polymers for Cancer Immunotherapy. Sci. Rep..

[147] Plebanek M.P., Bhaumik D., Bryce P.J., Thaxton C.S. (2018). Scavenger Receptor Type B1 and Lipoprotein Nanoparticle Inhibit Myeloid-Derived Suppressor Cells. Mol. Cancer Ther..

[148] Park J., Wrzesinski S.H., Stern E., Look M., Criscione J., Ragheb R., Jay S.M., Demento S.L., Agawu A., Licona Limon P. (2012). Combination delivery of TGF-beta inhibitor and IL-2 by nanoscale liposomal polymeric gels enhances tumour immunotherapy. Nat. Mater..

[149] Xu Z., Wang Y., Zhang L., Huang L. (2014). Nanoparticle-delivered transforming growth factor-beta siRNA enhances vaccination against advanced melanoma by modifying tumor microenvironment. ACS Nano.

[150] Ye Y., Wang J., Hu Q., Hochu G.M., Xin H., Wang C., Gu Z. (2016). Synergistic Transcutaneous Immunotherapy Enhances Antitumor Immune Responses through Delivery of Checkpoint Inhibitors. ACS Nano.

[151] Lu J., Liu X., Liao Y.P., Salazar F., Sun B., Jiang W., Chang C.H., Jiang J., Wang X., Wu A.M. (2017). Nano-enabled pancreas cancer immunotherapy using immunogenic cell death and reversing immunosuppression. Nat. Commun..

[152] Alhallak K., Sun J., Muz B., Jeske A., O’Neal J., Ritchey J.K., Achilefu S., DiPersio J.F., Azab A.K. (2022). Liposomal phytohemagglutinin: In vivo T-cell activator as a novel pan-cancer immunotherapy. J. Cell Mol. Med..

[153] Li S.Y., Liu Y., Xu C.F., Shen S., Sun R., Du X.J., Xia J.X., Zhu Y.H., Wang J. (2016). Restoring anti-tumor functions of T cells via nanoparticle-mediated immune checkpoint modulation. J. Control. Release Off. J. Control. Release Soc..

[154] Emens L.A., Cruz C., Eder J.P., Braiteh F., Chung C., Tolaney S.M., Kuter I., Nanda R., Cassier P.A., Delord J.P. (2019). Long-term Clinical Outcomes and Biomarker Analyses of Atezolizumab Therapy for Patients With Metastatic Triple-Negative Breast Cancer: A Phase 1 Study. JAMA Oncol..

[155] Schmid P., Adams S., Rugo H.S., Schneeweiss A., Barrios C.H., Iwata H., Dieras V., Hegg R., Im S.A., Shaw Wright G. (2018). Atezolizumab and Nab-Paclitaxel in Advanced Triple-Negative Breast Cancer. N. Engl. J. Med..

[156] West H., McCleod M., Hussein M., Morabito A., Rittmeyer A., Conter H.J., Kopp H.G., Daniel D., McCune S., Mekhail T. (2019). Atezolizumab in combination with carboplatin plus nab-paclitaxel chemotherapy compared with chemotherapy alone as first-line treatment for metastatic non-squamous non-small-cell lung cancer (IMpower130): A multicentre, randomised, open-label, phase 3 trial. Lancet. Oncol..

[157] Giannatempo P., Raggi D., Marandino L., Bandini M., Fare E., Calareso G., Colecchia M., Gallina A., Ross J.S., Alessi A. (2020). Pembrolizumab and nab-paclitaxel as salvage therapy for platinum-treated, locally advanced or metastatic urothelial carcinoma: Interim results of the open-label, single-arm, phase II PEANUT study. Ann. Oncol. Off. J. Eur. Soc. Med. Oncol..

[158] Morgensztern D., Dols M.C., Ponce Aix S., Postmus P.E., Bennouna J., Fischer J.R., Juan-Vidal O., Stewart D.J., Ardizzoni A., Bhore R. (2020). nab-Paclitaxel Plus Durvalumab in Patients with Previously Treated Advanced Stage Non-small Cell Lung Cancer (ABOUND.2L+). Front. Oncol..

[159] Bonvalot S., Rutkowski P.L., Thariat J., Carrere S., Ducassou A., Sunyach M.P., Agoston P., Hong A., Mervoyer A., Rastrelli M. (2019). NBTXR3, a first-in-class radioenhancer hafnium oxide nanoparticle, plus radiotherapy versus radiotherapy alone in patients with locally advanced soft-tissue sarcoma (Act.In.Sarc): A multicentre, phase 2–3, randomised, controlled trial. Lancet Oncol..

[160] Trujillo-Alonso V., Pratt E.C., Zong H., Lara-Martinez A., Kaittanis C., Rabie M.O., Longo V., Becker M.W., Roboz G.J., Grimm J. (2019). FDA-approved ferumoxytol displays anti-leukaemia efficacy against cells with low ferroportin levels. Nat. Nanotechnol..

